# Whole-genome sequencing-based characterization of antimicrobial resistance determinants and phenotype–genotype concordance in uropathogenic *Escherichia coli* isolates from ICU patients in Saudi Arabia

**DOI:** 10.3389/fmicb.2026.1931489

**Published:** 2026-10-30

**Authors:** Nada Almebairik, Duaa Bukhari, Ohoud Alhumaidan, Enaam Idrees, Haifa Asiri, Fuad Alanazi, Meyad Alkarni, Khaloud Alarjani, Basmah Almaarik

**Affiliations:** 1 Department of Clinical Laboratory Sciences, College of Applied Medical Sciences, King Saud University, Riyadh, Saudi Arabia; 2 Center of Excellence in Biotechnology Research, King Saud University, Riyadh, Saudi Arabia; 3 Microbiology Unit, Central Research Laboratory, King Saud University, Riyadh, Saudi Arabia; 4 Department of Botany and Microbiology, College of Science, King Saud University, Riyadh, Saudi Arabia

**Keywords:** antimicrobial resistance, extended-spectrum β-lactamase, phenotype–genotype concordance, Saudi Arabia, urinary tract infection, Uropathogenic *Escherichia coli*, whole-genome sequencing

## Abstract

*Escherichia coli* is a leading cause of urinary tract infections (UTIs) and an important reservoir of antimicrobial resistance (AMR). Whole-genome sequencing (WGS) enables comprehensive characterization of bacterial population structure and AMR determinants; however, limited data are available on the ability of WGS-derived resistance determinants to predict phenotypic AMR among urinary *E. coli* isolates. This study aimed to characterize the population structure and AMR determinants of *E. coli* isolates recovered from urine samples of intensive care unit (ICU) patients at a tertiary-care hospital in Riyadh, Saudi Arabia, and to evaluate phenotype–genotype concordance at the individual antimicrobial level. A total of 36 non-duplicate uropathogenic *E. coli* (UPEC) isolates recovered from urine samples of ICU patients between 2024 and 2025 underwent phenotypic antimicrobial susceptibility testing (AST) and WGS using the DNBSEQ-G400 platform. Phenotypic non-susceptibility was highest to ampicillin (100%), followed by cefuroxime and cefotaxime (94.4% each). All isolates were susceptible to imipenem, meropenem, and tigecycline, while one isolate was intermediate to amikacin. Extended-spectrum β-lactamase (ESBL) production was detected in 88.9% of isolates, and 94.4% exhibited a multidrug-resistant (MDR) phenotype. Genomic analysis identified 17 sequence types (STs), with ST131 and ST1193 being the most frequent lineages. bla_CTX-M_ genes were detected in 80.6% of isolates, predominantly bla_CTX-M-15_, and resistance-associated chromosomal mutations in the quinolone resistance-determining regions (QRDRs) were identified in 83.3%. Based on resistance determinants representing three or more antimicrobial categories, 97.2% of isolates exhibited a genotypic MDR profile. Overall phenotype–genotype concordance across individual antimicrobial agents was 90.9%, with high concordance for cefuroxime and cefotaxime (97.2% each), followed by gentamicin and levofloxacin (94.4% each), and low concordance for cefepime and norfloxacin (72.2% each). These findings demonstrate a high burden of AMR among genetically diverse *E. coli* isolates from a tertiary-care hospital in Riyadh and show that the ability of WGS-derived resistance determinants to predict phenotypic antimicrobial non-susceptibility varies across individual antimicrobial agents.

## 1 Introduction

*Escherichia coli* is a common commensal of the human gastrointestinal tract but is also the leading cause of UTIs and a major cause of other extraintestinal infections (Bok et al., 2025; Tawfick et al., 2022). Its ability to transition from a benign gut inhabitant to a uropathogen or an invasive bloodstream pathogen reflects a combination of ecological adaptability and genomic plasticity (Bok et al., 2025; Connor et al., 2023; Thänert et al., 2022; Hall et al., 2021). Advances in WGS have transformed the study of *E. coli*, enabling high-resolution analyses of population structure, AMR determinants, and molecular epidemiology (Shaik et al., 2022).

*E. coli* possesses a large and dynamic pangenome comprising a conserved core genome and a highly variable accessory genome. While the core genome reflects the evolutionary relationships among lineages and STs, the accessory genome shapes bacterial adaptation through the acquisition of AMR genes, virulence factors, and other fitness-associated traits (Chauhan et al., 2025; Cummins et al., 2022; Maurizi et al., 2025). Because AMR genes can disseminate across genetically distinct lineages through horizontal gene transfer, resistance profiles may not always reflect phylogenetic relationships (Tao et al., 2022; Whelan et al., 2024). Therefore, comprehensive characterization of AMR requires both phenotypic antimicrobial susceptibility testing and WGS to define lineage structure and identify resistance determinants (Tao et al., 2022; Whelan et al., 2024).

The clinical implications of this genomic flexibility are exemplified by extraintestinal pathogenic *E. coli* (ExPEC), which comprises strains capable of causing infections outside the gastrointestinal tract, including UTIs, bloodstream infections, and sepsis (Bok et al., 2025; Connor et al., 2023; Thänert et al., 2022). Within ExPEC, UPEC is the leading cause of UTIs worldwide (Maurizi et al., 2025; Huang et al., 2022). UPEC is also an important cause of healthcare-associated UTIs (Maurizi et al., 2025; Naziri et al., 2021). Patients admitted to ICUs are particularly susceptible to these infections because of prolonged hospitalization, indwelling urinary catheterization, and invasive procedures (Al-Amri et al., 2025; Yajun et al., 2025). Several internationally disseminated high-risk UPEC lineages have been identified, including ST131 and the emerging high-risk lineage ST1193, both of which are frequently associated with ESBL production and multidrug resistance (Nguyen et al., 2021; Kocsis et al., 2022; Manges et al., 2019). Together, these clinical and microbiological characteristics underscore the importance of further investigating UPEC isolates recovered from urine samples of ICU patients, particularly in regions where molecular epidemiological data remain limited.

Despite the increasing application of WGS in UPEC, important knowledge gaps remain. Several studies have combined phenotypic antimicrobial susceptibility testing with WGS to characterize resistance determinants and population structure; however, comparable data from clinical UPEC isolates in Saudi Arabia, particularly those originating from intensive care settings, remain limited (Whelan et al., 2024; Jackson et al., 2022). Furthermore, although internationally disseminated high-risk clones are recognized as major drivers of multidrug resistance, the contribution of genetically diverse STs to the local epidemiology of MDR UPEC remains incompletely understood, particularly in regions where genomic surveillance is limited (Quino et al., 2026; Byarugaba et al., 2023). Finally, accurate interpretation of genomic resistance determinants requires integration with phenotypic susceptibility testing, as phenotype–genotype concordance may vary across antimicrobial agents (Whelan et al., 2024; Jackson et al., 2022).

In this study, we characterized the population structure and AMR determinants of UPEC isolates recovered from urine samples of ICU patients at a tertiary-care hospital in Riyadh, Saudi Arabia. We also evaluated phenotype–genotype concordance to determine the ability of WGS-derived resistance determinants to predict phenotypic antimicrobial non-susceptibility across individual antimicrobial agents.

## 2 Methods

### 2.1 Study design and bacterial isolates

This retrospective laboratory-based study initially evaluated 40 non-duplicate *E. coli* isolates recovered from urine samples of ICU patients with clinically diagnosed UTIs at King Fahad Medical City (KFMC), Riyadh, Saudi Arabia, between 2024 and 2025. Urine specimens were processed according to standard clinical microbiology procedures, and the isolates were identified as *E. coli* using the Phoenix™ 100 Automated Microbiology System (Becton Dickinson Co., Sparks, MD, United States). Only the first *E. coli* isolate recovered from each patient with complete phenotypic antimicrobial susceptibility testing data was included. Duplicate isolates and isolates with incomplete phenotypic antimicrobial susceptibility testing data were excluded. Isolates were preserved at −80 °C in appropriate cryoprotective media until further phenotypic and genomic analyses were performed. The study was approved by the King Fahad Medical City Institutional Review Board (IRB No. 19-514E). Owing to the retrospective study design and the use of anonymized clinical isolates and laboratory data, the requirement for informed consent was waived.

### 2.2 Antimicrobial susceptibility testing and phenotypic resistance characterization

Antimicrobial susceptibility testing (AST) of *E. coli* isolates was performed using the Phoenix™ 100 Automated Microbiology System (Becton Dickinson Co., Sparks, MD, United States) based on automated broth microdilution methodology. The antimicrobial agents evaluated are listed in Table 1. Minimum inhibitory concentrations (MICs) and categorical interpretations (susceptible, intermediate, and resistant) were determined automatically. The results were interpreted according to the Clinical and Laboratory Standards Institute (CLSI) M100, 35th edition, 2025, where applicable (Clinical and Laboratory Standards Institute, 2025). ESBL production was inferred using the Phoenix™ ESBL interpretive algorithm based on MIC pattern analysis.

**Table 1:** Antimicrobial non-susceptibility rates and 95% confidence intervals among the 36 *E. coli iso*lates.

Antimicrobial category	Antimicrobial agent	Resistant, *n* (%)	Intermediate, *n* (%)	Non-susceptible, *n* (%)	95% CI
Penicillins	Ampicillin	36 (100.0)	0 (0.0)	36 (100.0)	90.4–100.0
Cephalosporins	Cefuroxime	34 (94.4)	0 (0.0)	34 (94.4)	81.9–98.5
	Cefotaxime	34 (94.4)	0 (0.0)	34 (94.4)	81.9–98.5
	Ceftazidime	20 (55.6)	10 (27.8)	30 (83.3)	68.1–92.1
	Cefepime	23 (63.9)	0 (0.0)	23 (63.9)	47.6–77.5
Carbapenems	Imipenem	0 (0.0)	0 (0.0)	0 (0.0)	0.0–9.6
	Meropenem	0 (0.0)	0 (0.0)	0 (0.0)	0.0–9.6
Fluoroquinolones	Levofloxacin	21 (58.3)	0 (0.0)	21 (58.3)	42.2–72.9
	Ciprofloxacin	21 (58.3)	3 (8.3)	24 (66.7)	50.3–79.8
	Norfloxacin	21 (58.3)	10 (27.8)	31 (86.1)	71.3–93.9
Folate pathway inhibitors	Trimethoprim-sulfamethoxazole	14 (38.9)	0 (0.0)	14 (38.9)	24.8–55.1
Aminoglycosides	Gentamicin	8 (22.2)	0 (0.0)	8 (22.2)	11.7–38.1
	Amikacin	0 (0.0)	1 (2.8)	1 (2.8)	0.5–14.2
Glycylcyclines	Tigecycline	0 (0.0)	0 (0.0)	0 (0.0)	0.0–9.6

CI, confidence interval; *n*, number of isolates.

Non-susceptible includes isolates classified as either intermediate or resistant.

Based on international consensus criteria, multidrug resistance (MDR) was defined as non-susceptibility to at least one agent in three or more antimicrobial categories (Magiorakos et al., 2012). In this study, the antimicrobial categories included penicillins, cephalosporins, carbapenems, fluoroquinolones, aminoglycosides, glycylcyclines, and folate pathway inhibitors. Non-susceptibility rates (%NS) were calculated as the proportion of isolates categorized as intermediate or resistant among those with valid susceptibility results for each antimicrobial agent.

### 2.3 Whole-genome sequencing

Genomic DNA was extracted from overnight cultures of *E. coli* grown in tryptic soy broth (TSB) using the QIAamp DNA Mini Kit (QIAGEN, Hilden, Germany) according to the manufacturer’s protocol. DNA was purified using a silica column-based extraction method and eluted in 30 μL of ultrapure water. DNA concentration and purity were evaluated using a NanoDrop 2000c spectrophotometer (Thermo Fisher Scientific, Waltham, MA, United States). DNA libraries were prepared using the MGIEasy FS DNA Library Prep Kit (MGI Tech Co., Ltd., China) according to the manufacturer’s instructions. Genomic DNA was sequenced using the DNBSEQ-G400 platform (MGI Tech Co., Ltd., Shenzhen, China) with 150 bp paired-end reads. Raw sequencing data were generated in FASTQ format for downstream bioinformatics analysis.

### 2.4 Bioinformatics analysis

Raw sequencing reads were assembled *de novo* using SPAdes (v3.9.0) with default parameters (Bankevich et al., 2012). Assembly quality was evaluated using SeqKit (v2.13.0) by calculating genome size, GC content, number of contigs, and N50, which were examined and reported as descriptive assembly-quality metrics. Genome completeness and contamination were further assessed using CheckM2 (v1.1.0; database version 3). Assemblies with ≥90% estimated completeness and ≤5% estimated contamination were considered to meet the primary CheckM2 quality criteria. Assemblies that did not meet either of these primary criteria were subjected to additional quality assessment rather than being automatically excluded. This assessment included evaluation of genome completeness and duplication using BUSCO (v6.0.0) with the Enterobacterales lineage dataset (enterobacterales_odb12) and investigation of potential foreign-sequence contamination through contig-level taxonomic classification using Kraken2 (v2.17.1; PlusPF database), followed by BLASTn (v2.16.0) analysis of larger contigs assigned to non-*Escherichia* taxa. Species identity was confirmed by average nucleotide identity (ANI) analysis using FastANI against the *E. coli* K-12 MG1655 reference genome. Isolates with ANI values of ≥95% were considered *E. coli*.

Phylogenetic groups (phylogroups) were assigned using ClermonTyping (v23.06) according to the Clermont *E. coli* phylogrouping scheme (Beghain et al., 2018). Multilocus sequence typing (MLST) was performed using the mlst software (v2.33.1) with the *E. coli* Achtman MLST scheme to assign STs. Allelic profiles were reviewed to confirm the successful identification of all seven housekeeping loci (*adk, fumC, gyrB, icd, mdh, purA,* and *recA*). Clonal complexes (CCs) were assigned using goeBURST via PHYLOViZ (https://online.phyloviz.net/index; accessed on 5 October 2026) (Ribeiro-Gonçalves et al., 2016). CCs were defined according to the eBURST criterion, whereby STs were assigned to the same CC when they were connected through single-locus variant (SLV) relationships, sharing six of the seven MLST loci with at least one other ST in the group (Feil et al., 2004). FimH types and serotypes were identified using FimTyper (v1.0) and SeroTypeFinder (v2.0), respectively, from the Center for Genomic Epidemiology (CGE) (https://www.genomicepidemiology.org/services/; accessed on 4 October 2026) (Roer et al., 2017; Joensen et al., 2015).

Acquired AMR genes and chromosomal mutations associated with fluoroquinolone resistance were identified using AMRFinderPlus (v4.2.7; database version 2026-05-15.1), with the organism parameter set to *Escherichia*. Acquired resistance genes were identified according to the curated detection criteria implemented in AMRFinderPlus (Feldgarden et al., 2021). Only complete acquired resistance gene calls and chromosomal substitutions classified by AMRFinderPlus as associated with fluoroquinolone resistance were considered AMR determinants, whereas acquired resistance gene hits classified as partial were excluded. For genotypic MDR classification, resistance determinants were assigned to the same antimicrobial categories used for phenotypic MDR classification according to their associated AMR class. A single resistance determinant could contribute to more than one antimicrobial category, where applicable. Isolates carrying resistance determinants associated with three or more of these antimicrobial categories were classified as having an MDR genotype (Magiorakos et al., 2012).

The core-genome alignment of the 36 *E. coli* isolates was generated using Parsnp (v2.1.5; https://github.com/marbl/parsnp), with the recombination-filtering option (-x) (Kille et al., 2024). Isolate KFMCEc17 (ST1193) was designated as the reference genome. This isolate was selected because it exhibited the highest assembly quality among the candidate representative isolates, with the lowest number of contigs and the highest N50, making it the most suitable reference for core-genome alignment. A maximum-likelihood phylogenetic tree was inferred using RAxML (v8.2.12) based on the recombination-filtered core-genome single-nucleotide polymorphism (SNP) alignment generated by Parsnp (Stamatakis, 2014). The resulting tree was annotated and visualized using the Interactive Tree of Life (iTOL) (v7.0; https://itol.embl.de; accessed on 18 September 2026).

The draft genome assemblies for all 36 isolates have been deposited in GenBank under BioProject accession number PRJNA1456941. BioSample and GenBank accession numbers, together with genome assembly-quality metrics and validation results for the 36 *E. coli* isolates, are provided in Supplementary Table S1.

### 2.5 Phenotype–genotype concordance analysis

Phenotype–genotype concordance was assessed at the individual antimicrobial level by comparing phenotypic susceptibility with resistance determinants identified using WGS. Determinant-to-antimicrobial associations were defined using AMRFinderPlus subclass annotations when specific antimicrobial agent associations were provided (Feldgarden et al., 2021). When AMRFinderPlus provided only broader antimicrobial class-level annotations, published literature was used to determine whether the identified determinant was associated with resistance to the individual antimicrobial agent tested (Husna et al., 2023; Jacoby et al., 2014; Poey et al., 2024; Rima et al., 2021; Rus et al., 2025; Tyson et al., 2015; Wu et al., 2014; Tewawong et al., 2025). The antimicrobial-specific resistance determinants and criteria used to define genotypic resistance (G+) in the phenotype–genotype concordance analysis are provided in Table 2.

**Table 2:** Antimicrobial-specific resistance determinants and criteria used to define genotypic resistance (G+) in the phenotype–genotype concordance analysis.

Antimicrobial agent	Determinants/Criteria defining G+	Supporting reference(s)
Amikacin	*aac(6′)-Ib-cr5*	AMRFinderPlus (Feldgarden et al., 2021)
Gentamicin	*aac(3)-IId, aac(3)-IIe, ant(2″)-Ia*	AMRFinderPlus (Feldgarden et al., 2021)
Ampicillin	*bla_TEM-1_, bla_OXA-1_,* bla_CTX-M_ family*, bla_CMY_* family*, bla_DHA-1_, bla_OXA-244_*	(Rima et al., 2021; Rus et al., 2025; Tyson et al., 2015)
Cefuroxime	bla_CTX-M_ family*, bla_CMY_* family*, bla_DHA-1_*	(Husna et al., 2023; Rus et al., 2025)
Cefotaxime	bla_CTX-M_ family*, bla_CMY_* family*, bla_DHA-1_*	(Husna et al., 2023; Rus et al., 2025)
Ceftazidime	bla_CTX-M_ family*, bla_CMY_* family*, bla_DHA-1_*	(Husna et al., 2023; Rus et al., 2025)
Cefepime	bla_CTX-M_ family	(Husna et al., 2023; Wu et al., 2014)
Imipenem	Carbapenemase determinant associated with imipenem resistance; *bla_OXA-244_* alone was not considered G + because of its reduced imipenem activity	(Rima et al., 2021)
Meropenem	Carbapenemase determinant associated with meropenem resistance; *bla_OXA-244_* alone was not considered G + because of its reduced meropenem activity	(Rima et al., 2021)
Ciprofloxacin	Presence of multiple QRDR substitutions, defined as *gyrA* S83L together with a second *gyrA* substitution at D87 (e.g., D87N or D87Y) and ≥1 resistance-associated *parC* substitution	(Jacoby et al., 2014; Tyson et al., 2015; Tewawong et al., 2025)
Levofloxacin	Presence of multiple QRDR substitutions, defined as *gyrA* S83L together with a second *gyrA* substitution at D87 (e.g., D87N or D87Y) and ≥1 resistance-associated *parC* substitution	(Jacoby et al., 2014; Tewawong et al., 2025)
Norfloxacin	Presence of multiple QRDR substitutions, defined as *gyrA* S83L together with a second *gyrA* substitution at D87 (e.g., D87N or D87Y) and ≥1 resistance-associated *parC* substitution	(Jacoby et al., 2014; Tewawong et al., 2025)
Trimethoprim-sulfamethoxazole	Concomitant presence of ≥1 *dfr* determinant and ≥1 *sul* determinant	(Poey et al., 2024)
Tigecycline	No applicable genotypic resistance determinant identified using the study’s AMRFinderPlus-based criteria.	Not applicable

G+, genotype-positive for resistance; QRDR, quinolone resistance-determining region; TMP–SMX, trimethoprim–sulfamethoxazole.

Resistance genes are shown according to AMRFinderPlus nomenclature.

Phenotypic results were categorized as susceptible (S) or non-susceptible (NS; intermediate or resistant). Phenotypic non-susceptibility (P+) was defined as non-susceptibility to the corresponding antimicrobial agent; otherwise, isolates were classified as phenotypically susceptible (P−). Isolates lacking phenotypic results for a given antimicrobial agent were excluded from the corresponding analysis. Genotypic resistance (G+) was assigned when the predefined antimicrobial-specific resistance determinant criterion was met; otherwise, isolates were classified as genotypically susceptible (G−). Two-by-two contingency tables were constructed for each antimicrobial agent to evaluate phenotype–genotype agreement.

### 2.6 Statistical analysis

This study employed descriptive statistical analyses using Python (v3.13.12). Categorical variables, including antimicrobial susceptibility results, STs, and AMR determinants, were summarized as frequencies and percentages. Non-susceptibility rates and phenotype–genotype performance measures, including sensitivity, specificity, positive predictive value (PPV), negative predictive value (NPV), and overall concordance (accuracy), were calculated for each antimicrobial agent. Corresponding 95% confidence intervals were calculated using the Wilson score method.

## 3 Results

### 3.1 Study isolates and selection

During the study period, 40 non-duplicate *E. coli* isolates were retrospectively recovered from urine specimens of ICU patients with clinically diagnosed UTIs at King Fahad Medical City (KFMC), Riyadh, Saudi Arabia, between 2024 and 2025. Four isolates were excluded because complete phenotypic antimicrobial susceptibility testing data were unavailable. Consequently, 36 isolates with complete antimicrobial susceptibility results were included in the study and underwent WGS and downstream genomic analyses.

### 3.2 Phenotypic antimicrobial susceptibility, ESBL production, and MDR

Across the tested agents, non-susceptibility was highest to ampicillin (100%), followed by cefuroxime and cefotaxime (both 94.4%). Non-susceptibility to ceftazidime and cefepime was observed in 83.3 and 63.9% of isolates, respectively. Fluoroquinolone non-susceptibility was also common, with 86.1% of isolates non-susceptible to norfloxacin, 66.7% to ciprofloxacin, and 58.3% to levofloxacin. Non-susceptibility to trimethoprim–sulfamethoxazole (TMP-SMX) was detected in 38.9% of isolates, whereas aminoglycoside non-susceptibility was less common, with 22.2% of isolates non-susceptible to gentamicin and 2.8% to amikacin. All isolates remained susceptible to imipenem, meropenem, and tigecycline. Complete antimicrobial susceptibility results with corresponding 95% confidence intervals are presented in Table 1.

Phenotypic antimicrobial susceptibility testing identified a high proportion of ESBL-producing and MDR isolates. Overall, 32 of the 36 isolates (88.9%) were classified as ESBL producers based on the Phoenix™ system. Phenotypic MDR was observed in 34 of the 36 isolates (94.4%). The median number of non-susceptible antimicrobial categories per isolate was 3.5 (IQR, 3–4; range, 1–5).

### 3.3 Genome assembly quality and sequence typing

Assembly quality was assessed for all 36 *E. coli* genome assemblies using SeqKit. Genome sizes ranged from 4.69 to 6.30 Mb, GC contents ranged from 50.28 to 50.85%, contig counts ranged from 190 to 1,273, and N50 values ranged from 18.3 to 262.4 kb. CheckM2 estimated 100% completeness for all assemblies, while estimated contamination ranged from 0 to 6.39%. Thirty-five of the 36 assemblies (97.2%) had estimated contamination ≤5%. KFMCEc01 was the only assembly exceeding this threshold, with an estimated contamination of 6.39%; this assembly also had the largest genome size (6.30 Mb), the highest number of contigs (1,273), and the lowest N50 (18.3 kb). Additional assessment of KFMCEc01 using BUSCO showed 98.7% complete BUSCOs, including 97.2% single-copy and 1.5% duplicated BUSCOs. Contig-level taxonomic classification using Kraken2 predominantly assigned sequences to Enterobacteriaceae/*Escherichia*, and BLASTn analysis of the larger contigs assigned to non-*Escherichia* taxa showed complete or near-complete query coverage and approximately 100% nucleotide identity to *E. coli* sequences. KFMCEc01 was therefore retained for downstream analyses.

Species identity was subsequently confirmed by ANI analysis using FastANI against the *E. coli* K-12 MG1655 reference genome. All assemblies exhibited ANI values above the 95% species-level threshold (96.65–99.36%), confirming their identification as *E. coli*. MLST was performed using the *E. coli* Achtman MLST scheme implemented in the mlst software (v2.33.1). Complete allelic profiles comprising the seven housekeeping loci (*adk, fumC, gyrB, icd, mdh, purA,* and *recA*) were successfully identified for all isolates. STs were assigned to 35 of the 36 isolates (97.2%), whereas one isolate (KFMCEc19) remained unassigned because its allelic profile did not correspond to a defined ST in the current Achtman MLST database. Genome assembly-quality metrics and validation results for the 36 *E. coli* isolates are summarized in Supplementary Table S1.

### 3.4 Phylogenetic groups and MLST

*In silico* MLST identified 17 distinct STs among the study isolates, with one isolate remaining unassigned. ST131 was the most frequently detected lineage (19.4%, 7/36), followed by ST1193 (11.1%, 4/36). ST73 and ST450 each accounted for 8.3% (3/36), whereas the remaining STs occurred at lower frequencies. Several STs identified in this study, including ST13262, ST15519, ST1431, ST12637, and ST10320, have received limited characterization in the published literature (Figure 1). Based on the eBURST criterion for CC definition, ST15519 and ST10 formed an SLV-linked CC, differing only at the *mdh* locus, whereas the unassigned ST profile was a double-locus variant (DLV) of ST10. Similarly, ST10320 and ST73 formed an SLV-linked CC, differing only at the *adk* locus, while ST13262 was an SLV of ST1193, differing only at the *gyrB* locus.

**Figure 1:**
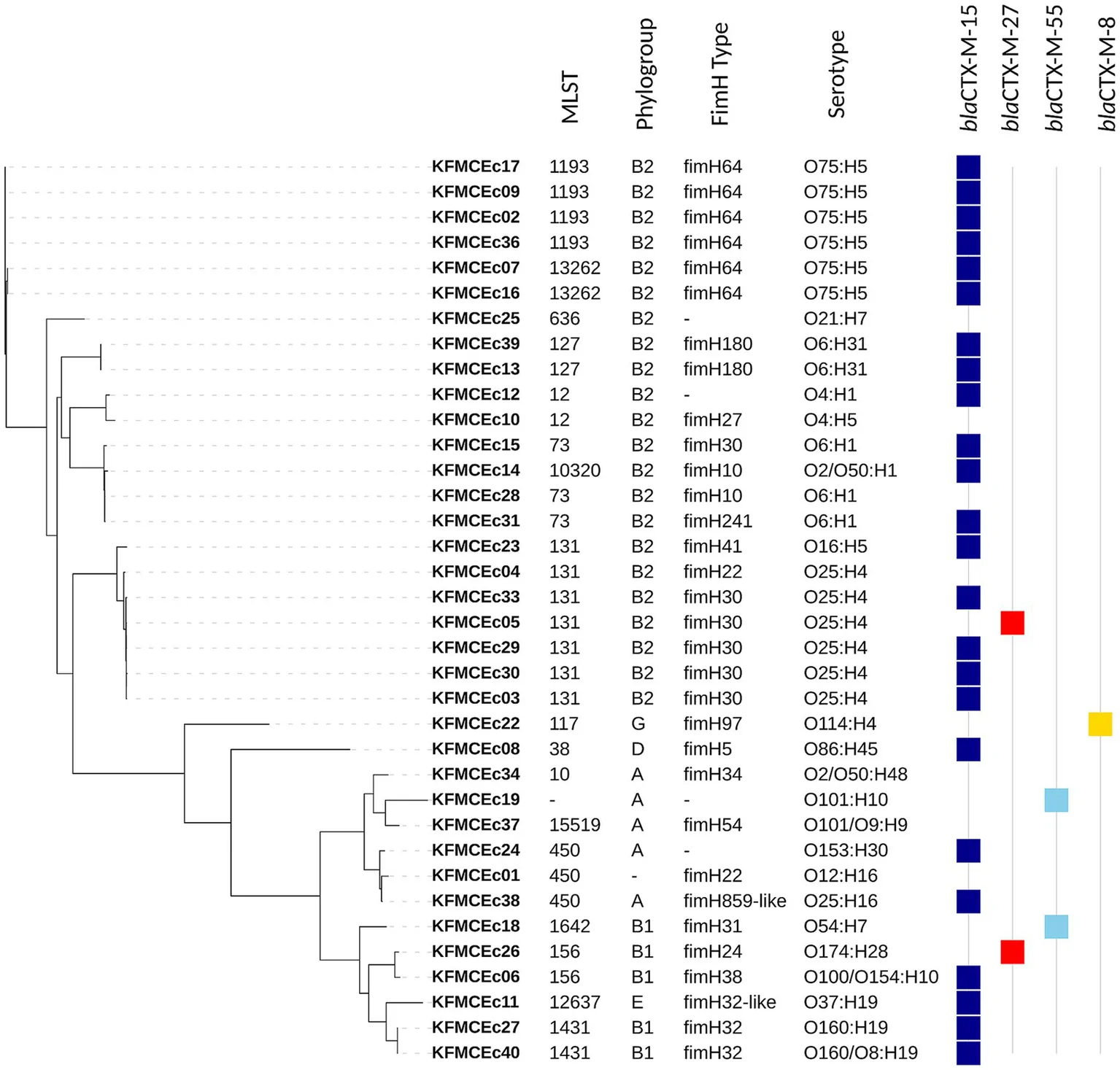
Core-genome SNP phylogeny of 36 uropathogenic *Escherichia coli iso*lates from ICU patients in Saudi Arabia. The maximum-likelihood phylogenetic tree was inferred using RAxML from the recombination-filtered core-genome SNP alignment generated by Parsnp. Branch lengths are proportional to evolutionary distance. *Annotation columns indicate MLST sequence type, phylogroup, FimH type, serotype, and the presence of bla_CTX-M-15_, bla_CTX-M-27_, bla_CTX-M-55_, and bla_CTX-M-8_ genes.

Phylogenetic grouping analysis revealed that phylogroup B2 was predominant, accounting for 61.1% of isolates (22/36), followed by phylogroups A and B1, each representing 13.9% (5/36). Phylogroups D, E, and G were each represented by a single isolate (2.8%, 1/36), while one isolate (2.8%, 1/36) could not be assigned to a phylogroup. The major lineages ST131, ST1193, and ST73, together with their SLVs ST13262 and ST10320, all belonged to phylogroup B2 (Figure 1).

### 3.5 Serotyping and FimH typing

Serotyping and FimH typing identified 26 distinct O: H-fimH profiles among the 36 isolates. For ST131 isolates, the predominant profile was O25: H4-*fimH30* (5/7), while single isolates were identified as O25: H4-*fimH22* and O16: H5-*fimH41*. All ST1193 isolates and their SLV, ST13262, exhibited the O75: H5-*fimH64* profile. ST73 isolates were uniformly O6: H1 but showed three different FimH types, namely, *fimH30*, *fimH10*, and *fimH241*, whereas the SLV ST10320 isolate carried *fimH10* with an ambiguous O2/O50: H1 serotype call. Serotype and FimH profiles among the remaining isolates varied across STs (Figure 1).

### 3.6 Phylogenetic clustering and lineage-associated profiles

Phylogenetic analysis showed clustering largely consistent with MLST and phylogroup assignments (Figure 1). The major B2 lineages ST131, ST1193, and ST73 formed distinct lineage-associated clusters. ST1193 isolates clustered closely with the ST1193 SLV ST13262 and shared the B2-O75: H5-*fimH64* profile. ST73 isolates clustered with the ST73-related SLV ST10320 while showing diversity in *FimH* type and serotype. ST131 isolates formed a closely related group dominated by the O25: H4-*fimH30* profile, with additional O25: H4-*fimH22* and O16: H5-*fimH41* profiles. The two ST1431 isolates clustered closely and shared phylogroup B1, *fimH32*, and an O160: H19-compatible serotype profile. The remaining STs were distributed across separate branches and showed heterogeneous phylogroup, serotype, and *FimH* profiles (Figure 1).

### 3.7 Distribution of AMR determinants

WGS-based analysis identified AMR determinants across multiple antimicrobial categories (Supplementary Table S2). β-Lactam resistance was dominated by the ESBL genes of the bla_CTX-M_ family, which were detected in 29 of the 36 isolates (80.6%), with bla_CTX-M-15_ representing the predominant allele and occurring across multiple STs, whereas other bla_CTX-M_ variants were detected infrequently. Additional β-lactam resistance determinants included AmpC β-lactamases (*bla_CMY_* and *bla_DHA_*) and penicillinase genes of the *bla_TEM_* family and *bla_OXA-1_*. The carbapenemase gene *bla_OXA-244_* was identified in only one isolate (KFMCEc25, ST636) (Table 3).

**Table 3:** Distribution of antimicrobial resistance determinants among the 36 *E. coli* isolates.

Antimicrobial category	Resistance determinant	Isolates, *n* (%)
ESBL	blaCTX-M (overall)	29 (80.6)
	*bla*_CTX-M-15_	24 (66.7)
	*bla*_CTX-M-55_	2 (5.6)
	*bla*_CTX-M-27_	2 (5.6)
	*bla*_CTX-M-8_	1 (2.8)
AmpC β-lactamase	*bla*_CMY_/*bla_DHA_*	5 (13.9)
Penicillinase	*bla_TEM_*/*bla_OXA-1_*	16 (44.4)
Carbapenemase	*bla_OXA-244_*	1 (2.8)
Aminoglycosides	*aac*, *aadA*, *ant*, *aph*	20 (55.6)
Fluoroquinolones (PMQR)	*qnrB4*, *qnrS1*, *qnrS13, aac*(6′)-Ib-cr5	20 (55.6)
Fluoroquinolones (QRDR mutations)	*gyrA, parC, and/or parE mutations*	30 (83.3)
Folate inhibitors	*dfrA*, *sul*	18 (50.0)
Tetracyclines	*tet(A), tet(B)*	15 (41.7)
Phenicols	*cat*, *floR*	4 (11.1)
Macrolide/Lincosamide	*mphA*, *mefB*, *lnu(F)*	17 (47.2)

ESBL, extended-spectrum β-lactamase; PMQR, plasmid-mediated quinolone resistance; QRDR, quinolone resistance-determining region; *n*, number of isolates.

Acquired resistance determinants associated with aminoglycosides, plasmid-mediated quinolone resistance (PMQR), and folate pathway inhibitors were also detected among the isolates (Table 3). In addition to acquired fluoroquinolone resistance genes, resistance-associated chromosomal mutations in the QRDRs of *gyrA*, *parC*, and/or *parE* were identified in 30 isolates (83.3%). Resistance determinants associated with tetracyclines, phenicols, and macrolide/lincosamide resistance were also detected. The distribution of AMR determinants is summarized in Table 3, and isolate-level profiles provided in Supplementary Table S2.

The genomic burden of AMR varied considerably among the isolates. Five isolates carried more than 15 AMR determinants, including three ST131 isolates (KFMCEc23, KFMCEc29, and KFMCEc33), one ST450 isolate (KFMCEc38), and one ST15519 isolate (KFMCEc37). Based on the presence of AMR determinants representing three or more antimicrobial categories, 35 of the 36 isolates (97.2%) were classified as having an MDR genotype. The number of AMR determinants per isolate ranged from 1 to 23, with a median of 8 (interquartile range [IQR], 3–13). The number of antimicrobial categories represented by at least one resistance determinant per isolate had a median of 4 (IQR, 3–5) and ranged from 1 to 5 (Supplementary Table S2).

### 3.8 Phenotype–genotype concordance and diagnostic performance

Phenotype–genotype concordance was evaluated by comparing resistance determinants identified using WGS with phenotypic antimicrobial susceptibility results for individual antimicrobial agents and with phenotypic ESBL and MDR classifications across the 36 isolates (Tables 2, 4). For the individual antimicrobial agents, overall concordance across all isolate–antimicrobial comparisons was 90.9%, although the degree of concordance varied among antimicrobial agents. After excluding ampicillin, imipenem, meropenem, and tigecycline, which showed little or no phenotypic variability, concordance was 87.2% (314/360).

**Table 4:** Phenotype–genotype concordance and diagnostic performance for individual antimicrobial agents and composite resistance classifications among the 36 *E. coli iso*lates.

Antimicrobial agent	TP	FP	FN	TN	Concordance% (95% CI)	Sensitivity%	Specificity%	PPV	NPV
Ampicillin	36	0	0	0	100 (90.4–100)	100	N/A	100	N/A
Cefuroxime	33	0	1	2	97.2 (85.8–99.5)	97.1	100	100	66.7
Cefotaxime	33	0	1	2	97.2 (85.8–99.5)	97.1	100	100	66.7
Ceftazidime	29	4	1	2	86.1 (71.3–93.9)	96.7	33.3	87.9	66.7
Cefepime	21	8	2	5	72.2 (56–84.2)	91.3	38.5	72.4	71.4
Imipenem	0	0	0	36	100 (90.4–100)	N/A	100	N/A	100
Meropenem	0	0	0	36	100 (90.4–100)	N/A	100	N/A	100
Gentamicin	8	2	0	26	94.4 (81.9–98.5)	100	92.9	80	100
Amikacin	1	7	0	28	80.6 (65–90.2)	100	80	12.5	100
Tigecycline	0	0	0	36	100 (90.4–100)	N/A	100	N/A	100
Trimethoprim-sulfamethoxazole	13	2	1	20	91.7 (78.2–97.1)	92.9	90.9	86.7	95.2
Ciprofloxacin	20	1	4	11	86.1 (71.3–93.9)	83.3	91.7	95.2	73.3
Levofloxacin	20	1	1	14	94.4 (81.9–98.5)	95.2	93.3	95.2	93.3
Norfloxacin	21	0	10	5	72.2 (56–84.2)	67.7	100	100	33.3
ESBL and MDR classification									
ESBL	29	0	3	4	91.7 (78.2–97.1)	90.6	100	100	57.1
MDR	34	1	0	1	97.2 (85.8–99.5)	100	50	97.1	100

Phenotypic non-susceptibility (R or I) was defined as a positive phenotypic result (P+), whereas phenotypic susceptibility (S) was defined as a negative phenotypic result (P−). Genotypic detection of a corresponding resistance determinant was defined as G+, and the absence of a corresponding resistance determinant was defined as G−. TP = P+/G+; FP = P−/G+; TN = P−/G−; and FN = P+/G−. Sensitivity = TP/(TP + FN); specificity = TN/(TN + FP); PPV = TP/(TP + FP); and NPV = TN/(TN + FN). NA indicates that the corresponding measure could not be calculated because the denominator was zero.

CI, confidence interval; FN, false negative; FP, false positive; NPV, negative predictive value; PPV, positive predictive value; TN, true negative; TP, true positive.

Complete concordance (100%) was observed for ampicillin, imipenem, meropenem, and tigecycline. For imipenem, meropenem, and tigecycline, all 36 isolates were phenotypically susceptible, and no individual-agent-specific resistance determinants were identified among the AMRFinderPlus-detected determinants, resulting in 100% concordance in this study. Among the cephalosporins, high concordance was observed for cefuroxime and cefotaxime (97.2% each). The single false-negative isolate for cefuroxime and cefotaxime contained a partial *bla*_CTX-M_ family hit at a contig boundary (72.51% coverage), which was excluded according to the predefined genotypic criteria. Concordance was lower for ceftazidime (86.1%) and cefepime (72.2%), with discordance primarily attributable to false-positive genotypic predictions (Table 4).

Among the aminoglycosides, concordance was 94.4% for gentamicin and 80.6% for amikacin. All phenotypically non-susceptible isolates carried at least one corresponding aminoglycoside resistance determinant; however, such determinants were also detected in two gentamicin-susceptible and seven amikacin-susceptible isolates, resulting in false-positive genotypic predictions and lower positive predictive values, particularly for amikacin. Concordance for trimethoprim–sulfamethoxazole was 91.7%. One false-negative isolate carried a complete *dfrA1* gene together with a partial *sul2* hit at a contig boundary (72.69% coverage); the latter was excluded according to the predefined genotypic criteria. In contrast, the two false-positive isolates carried complete *dfrA* and *sul* genes, indicating that discordance reflected both the exclusion of a partial resistance determinant and the presence of complete resistance determinants in phenotypically susceptible isolates. Among the fluoroquinolones, concordance was highest for levofloxacin (94.4%), followed by ciprofloxacin (86.1%) and norfloxacin (72.2%) (Table 4).

For the composite resistance classifications, phenotype–genotype concordance was 91.7% for ESBL and 97.2% for MDR. For ESBL, 29 isolates were concordantly classified as positive, three phenotypically ESBL-positive isolates were genotypically negative, and four isolates were negative by both approaches. For MDR, 34 isolates were concordantly classified as MDR and one isolate as non-MDR, while one phenotypically non-MDR isolate was classified as genotypically MDR (Table 4).

## 4 Discussion

This study combined phenotypic antimicrobial susceptibility testing and WGS to characterize the AMR profiles, population structure, and genomic diversity of uropathogenic *E. coli* isolates recovered from ICU patients at a tertiary-care hospital in Riyadh, Saudi Arabia. These findings demonstrate a substantial burden of AMR, characterized by a high proportion of ESBL-producing and MDR isolates, multiple acquired resistance determinants, and the circulation of multiple internationally recognized high-risk clones. Phenotype–genotype concordance was high for most antimicrobial agents, although reduced concordance for selected agents highlighted the limitations in predicting phenotypic resistance from the detection of resistance determinants alone.

The phenotypic antimicrobial susceptibility profile demonstrated high levels of non-susceptibility to several antimicrobial agents among the study isolates. Complete non-susceptibility to ampicillin was observed, together with very high non-susceptibility to cefuroxime and cefotaxime and substantial non-susceptibility to ceftazidime, cefepime, and fluoroquinolones. These findings are consistent with the high proportion of phenotypic ESBL production observed in this study. Similar high rates of ESBL production and multidrug resistance have been reported among healthcare-associated UPEC isolates from Saudi Arabia and other countries (Aljohani et al., 2023; Al-Agamy et al., 2014; Alyamani et al., 2017; Alqasim et al., 2018; Alreshidi, 2025; Das et al., 2026; Al-Mufarji et al., 2026; Zeng et al., 2025; Wu et al., 2025; Pitout and Chen, 2023). In contrast, carbapenems, tigecycline, and amikacin retained high *in vitro* activity against the isolates, consistent with previous reports of preserved susceptibility to these agents (Aljohani et al., 2023; Alqasim et al., 2018; Das et al., 2026; Kot et al., 2021).

The observed phenotypic resistance profile was supported by the WGS findings, which identified ESBL genes in 29 of the 36 isolates. Among the acquired AMR determinants identified, bla_CTX-M-15_ was the predominant ESBL gene, accompanied by additional β-lactamase determinants, including penicillinases and acquired AmpC enzymes. The predominance of bla_CTX-M-15_ closely reflects previous national and international reports identifying this gene as the predominant ESBL determinant among UPEC isolates and is consistent with the high non-susceptibility to the third-generation cephalosporins observed among the study isolates (Jackson et al., 2022; Aljohani et al., 2023; Alqasim et al., 2018; Zeng et al., 2025; Tsui et al., 2024; Alangari et al., 2022).

Further *in silico* investigation was performed on the generated sequences to characterize the population structure of the study isolates. MLST analysis identified 17 distinct STs among the 35 isolates with assigned STs, indicating the circulation of multiple lineages, including internationally recognized high-risk lineages and less well-characterized STs. ST131 was the predominant UPEC lineage, followed by ST1193 and ST73; the majority of isolates belonging to these major lineages exhibited ESBL production and multidrug resistance. The predominance of ST131 is consistent with previous studies from Saudi Arabia and international reports identifying this lineage, particularly the O25: H4-*fimH30* sub-lineage, as a major high-risk clone associated with ESBL production and multidrug resistance (Aljohani et al., 2023; Zeng et al., 2025; Price et al., 2013; Nicolas-Chanoine et al., 2014). In agreement with these observations, the majority of ST131 isolates in the present study exhibited the O25: H4-*fimH30* profile. Similarly, ST1193, associated with the O75: H5-*fimH64* profile, has emerged as a globally disseminated UPEC lineage and is increasingly recognized as an important high-risk clone associated with fluoroquinolone resistance and CTX-M-type ESBL production (Pitout et al., 2022; García-Meniño et al., 2024; Fukushima et al., 2021; Johnson et al., 2019). ST73 is a well-recognized, internationally disseminated UPEC lineage commonly associated with the O6: H1 serotype and has been repeatedly identified among urinary isolates in Saudi Arabia. Although it is not primarily characterized as a globally dominant MDR clone such as ST131 or ST1193, MDR- and ESBL-producing ST73 isolates have increasingly been reported (Aljohani et al., 2023; Bogema et al., 2020; de Souza da-Silva et al., 2017; Li et al., 2023).

MLST allelic relationships additionally showed that several less common STs were closely associated with established lineages at the MLST level. ST13262, ST10320, and ST15519 were SLVs of ST1193, ST73, and ST10, respectively. The unassigned ST profile was a DLV of ST10, suggesting broader allelic relatedness while remaining outside the SLV-based clonal-complex definition (Feil et al., 2004). These findings indicate that some of the less frequently encountered STs in this study were associated with established *E. coli* lineages based on the population structure analysis, rather than representing completely unrelated genetic backgrounds. However, ST13262, ST15519, ST1431, ST12637, and ST10320 remain comparatively poorly characterized in the published literature, and their low frequency in this small collection limits conclusions regarding their prevalence or epidemiological significance, emphasizing the need for larger, multicenter genomic studies.

The phylogroup assignment further showed that the predominant UPEC lineages ST131, ST1193, and ST73 all belonged to phylogroup B2, whereas the remaining STs were distributed across phylogroups A, B1, D, E, and G. The predominance of phylogroup B2 in the present study is consistent with previous UPEC findings from Saudi Arabia, in which B2 was similarly the dominant phylogenetic group among the detected phylogroups (Aljohani et al., 2023).

In addition to characterizing population structure, WGS demonstrated high overall phenotype–genotype concordance, particularly for antimicrobial agents for which resistance is primarily mediated by well-characterized resistance determinants. Complete concordance was observed for ampicillin, while high concordance was observed for cefuroxime and cefotaxime, reflecting the close correspondence between acquired β-lactamase genes and phenotypic non-susceptibility. The single false-negative prediction for cefuroxime and cefotaxime may partly reflect the conservative exclusion of a partial *bla*_CTX-M_ family call located at a contig boundary, which may represent incomplete gene recovery associated with the draft genome assembly (Feldgarden et al., 2021). In contrast, concordance for ceftazidime and cefepime was lower, with several isolates carrying determinants meeting the predefined genotypic resistance criteria for these agents but remaining phenotypically susceptible. Similar variability in cephalosporin susceptibility among ESBL-positive *E. coli* has been reported previously; Wu et al. (2023) found substantially lower resistance to ceftazidime (41.3%) and cefepime (35.4%) than to cefotaxime (100%) among ESBL-positive isolates. These findings indicate that the presence of ESBL determinants is not consistently associated with phenotypic resistance to individual cephalosporins and that resistance phenotypes may be influenced by the specific β-lactamase genotype and additional mechanisms, including altered membrane permeability, efflux activity, and regulatory effects, as well as differences in the genetic context and expression of *bla*_CTX-M_ genes (Wu et al., 2023; Nasrollahian et al., 2024; Peirano et al., 2025). Future studies incorporating targeted analysis of *bla*_CTX-M_ regulatory regions and expression, together with long-read sequencing to resolve the plasmid and genomic context of resistance determinants, may help clarify these genotype–phenotype discrepancies.

Carbapenem phenotype–genotype analysis showed complete concordance for imipenem and meropenem, with all isolates classified as phenotypically susceptible and genotypically susceptible at the individual-agent level. Although a single phenotypically susceptible isolate carried *bla*_OXA-244_, this carbapenemase determinant was not considered sufficient to define genotypic resistance to imipenem or meropenem. Similar observations have been reported for OXA-244-producing *E. coli*, in which reduced carbapenem-hydrolyzing activity can result in low carbapenem MICs despite the presence of *bla*_OXA-244_, making these isolates difficult to detect using routine phenotypic susceptibility testing (Rima et al., 2021; Nasrollahian et al., 2024; Notermans et al., 2022; Sattler et al., 2025). In addition, carbapenem resistance is influenced by multiple bacterial factors, including carbapenemase expression levels, gene copy number, enzyme activity, alterations in outer membrane permeability, and other regulatory mechanisms (Rima et al., 2021; Nasrollahian et al., 2024; Notermans et al., 2022; Sattler et al., 2025). Therefore, the presence of *bla*_OXA-244_ alone may not be sufficient to confer phenotypically detectable carbapenem resistance. For tigecycline, classical *tet* genes were detected despite universal phenotypic susceptibility to this agent. Previous studies have shown that tigecycline resistance is more commonly associated with specific *tet(A)* variants, *tet(X)* enzymes, efflux pump overexpression, or regulatory mutations than with the presence of other *tet* genes alone (Wang and Li, 2024; Li et al., 2023; Korczak et al., 2024).

The variability in phenotype–genotype concordance for levofloxacin, ciprofloxacin, and norfloxacin highlights the complexity of fluoroquinolone resistance mechanisms. In *E. coli,* fluoroquinolone resistance is primarily mediated by chromosomal mutations within the QRDRs of *gyrA* and *parC* and less frequently *gyrB* or *parE*, together with PMQR determinants (Tewawong et al., 2025; Amel et al., 2024; Osińska et al., 2025). The accumulation of QRDR mutations is generally associated with higher levels of fluoroquinolone resistance, whereas PMQR determinants typically confer lower-level resistance and may act in combination with chromosomal mutations (Tewawong et al., 2025; Amel et al., 2024; Osińska et al., 2025). In addition, mechanisms such as altered efflux and permeability may contribute to fluoroquinolone resistance but were not evaluated in the present study (Tewawong et al., 2025; Amel et al., 2024). Therefore, the detected QRDR mutations and acquired resistance determinants may not fully account for phenotypic resistance to individual fluoroquinolones.

For trimethoprim–sulfamethoxazole, phenotype–genotype concordance was high, although discordance occurred in both directions. Isolates non-susceptible to TMP-SMX carried *sul* genes in combination with *dfrA* genes, a resistance profile commonly reported among MDR *E. coli* isolates (Poey et al., 2024; Zeng et al., 2025; Grevskott et al., 2021). The single false-negative prediction in the present study carried a complete *dfrA1* gene but had a partial *sul2* call located at a contig boundary that was excluded according to the predefined genotypic criteria (Feldgarden et al., 2021). Conversely, two phenotypically susceptible isolates carried complete *dfr* and *sul* determinants, indicating that the presence of both resistance gene types was not invariably associated with phenotypic non-susceptibility. The expression of *dfrA* genes can depend on their genetic context and promoter architecture, suggesting that gene detection alone may not fully capture the resulting resistance phenotype (Poey et al., 2024). These findings highlight the importance of considering both sequence completeness and the genetic context of resistance determinants when interpreting WGS-based predictions of trimethoprim–sulfamethoxazole resistance.

Phenotype–genotype concordance for aminoglycosides was high for gentamicin but lower for amikacin, primarily due to genotype-positive/phenotype-susceptible discordance observed for both agents. Although genotypic resistance was defined using antimicrobial-specific resistance determinants, the presence of these determinants was not invariably associated with phenotypic non-susceptibility. Previous studies have similarly demonstrated that the detection of aminoglycoside-modifying enzyme genes does not always correspond directly to the observed aminoglycoside resistance phenotype (Zhang et al., 2023; Ghamari et al., 2024). This discordance may reflect factors beyond gene presence alone, including differences in gene expression, functional activity, and the broader genomic context of the aminoglycoside resistance determinants. Future studies incorporating expression analyses, together with sequencing approaches capable of resolving the plasmid and mobile genetic element context of aminoglycoside resistance determinants, may provide further insight into the mechanisms underlying the observed phenotype–genotype discordance. Overall, the variation in phenotype–genotype concordance observed across the antimicrobial agents evaluated in this study indicates that the detection of a resistance determinant does not necessarily correspond to phenotypic non-susceptibility. Such discordance may reflect the complexity of phenotype–genotype relationships and the contribution of multiple interacting resistance mechanisms (James et al., 2025; Hu et al., 2024).

This distinction is also relevant when comparing phenotypic MDR and genotypic MDR. Phenotypic MDR was defined as non-susceptibility to at least one agent in three or more antimicrobial categories, whereas genotypic MDR was based on the presence of resistance determinants assigned to these categories (Magiorakos et al., 2012). The slightly higher prevalence of genotypic MDR than phenotypic MDR may therefore reflect, at least in part, the different bases of these classifications. Because some resistance determinants have agent-specific effects, category-level detection of a resistance determinant may indicate resistance potential without necessarily corresponding to phenotypic non-susceptibility to the specific antimicrobial agent tested within that category. This represents an inherent interpretive limitation of the genotypic MDR metric and indicates that genotypic MDR and phenotypic MDR estimates should not be considered directly interchangeable.

Taken together, the findings of this study have implications for AMR surveillance. The high proportion of ESBL-producing and MDR UPEC isolates highlights the importance of continued genomic and phenotypic surveillance to monitor resistance patterns and characterize circulating resistant lineages (Hu et al., 2024; World Health Organization, 2025; Watt et al., 2025). Similar findings have been reported among UPEC isolates from Saudi Arabia and other countries, supporting the value of continued surveillance of AMR in this clinically important pathogen (Aljohani et al., 2023; Al-Agamy et al., 2014; Alyamani et al., 2017; Alqasim et al., 2018; Alreshidi, 2025; Das et al., 2026; Al-Mufarji et al., 2026; Zeng et al., 2025; Wu et al., 2025). The presence of internationally recognized high-risk clones together with the identification of less frequently reported STs further highlights the diverse population structure of UPEC and reinforces the value of WGS-based surveillance for monitoring clinically important lineages (Watt et al., 2025; Geurtsen et al., 2022). Within Saudi Arabia and the wider GCC region, broader implementation of genomic surveillance could complement existing AMR surveillance programs by providing higher-resolution characterization of circulating clones, resistance determinants, and their distribution (Aljohani et al., 2023; Alreshidi, 2025; Tsui et al., 2024).

Several limitations should be considered when interpreting these findings. First, this was a single-center study involving a relatively small number of isolates, which may have limited the precision of the estimates and may affect the generalizability of the findings. Therefore, the results should be interpreted with caution and validated in larger, multicenter studies. Second, only urinary isolates recovered from ICU patients were included; therefore, the findings may not represent the broader population of UPEC causing community-acquired or non-ICU healthcare-associated infections. Third, the genomic analyses focused primarily on AMR determinants and population structure. Virulence-associated genomic features were not investigated, limiting assessment of the relationship between virulence potential and AMR. Finally, detailed patient-level clinical and epidemiological data, including demographic characteristics, underlying conditions, catheterization status, prior antimicrobial exposure, duration of ICU stay, treatment, and clinical outcomes, were not available. This limited the assessment of associations between AMR or genomic characteristics and clinical factors or outcomes, as well as the investigation of potential transmission events or clonal dissemination within the ICU.

An additional limitation concerns the precision of the antimicrobial agent-specific genotype–phenotype performance estimates. Sparse cells in several contingency tables made some estimates sensitive to the classification of individual isolates. This was particularly evident for amikacin, for which the PPV of 12.5% was based on one true-positive and seven false-positive isolates, while sensitivity was determined by a single phenotypically non-susceptible isolate. Similarly, for ceftazidime, the presence of only one false-negative and two true-negative isolates limited the precision of the specificity and NPV estimates. For cefuroxime and cefotaxime, although the single false-negative may partly reflect the conservative exclusion of a partial *bla*_CTX-M_ family call at a contig boundary, the small number of genotype-negative isolates also limits the precision of the corresponding NPV estimates. These antimicrobial agent-specific estimates should therefore be interpreted cautiously and confirmed in larger isolate collections.

In conclusion, this study used both phenotypic antimicrobial susceptibility testing and WGS to characterize the AMR profiles and population structure of UPEC isolates from ICU patients in Saudi Arabia. The results demonstrate a high proportion of ESBL-producing and MDR UPEC, substantial genetic diversity, the presence of internationally disseminated high-risk clones, and high phenotype–genotype concordance for several antimicrobial agents. Collectively, these results highlight the complementary information provided by phenotypic antimicrobial susceptibility testing and WGS for characterizing AMR and provide a foundation for future multicenter genomic studies in Saudi Arabia. Given the limited sample size and lack of detailed patient-level data, these findings should be interpreted primarily in the context of genomic and AMR characterization. Future multicenter studies integrating AMR profiling with analyses of virulence determinants, plasmid characterization, horizontal gene transfer, and detailed clinical data could provide a more comprehensive understanding of the epidemiology and clinical significance of UPEC infections.

## Data Availability

The datasets presented in this study can be found in online repositories. The names of the repository/repositories and accession number(s) can be found in the article/Supplementary material.
